# ANGLOR: A Composite Machine-Learning Algorithm for Protein Backbone Torsion Angle Prediction

**DOI:** 10.1371/journal.pone.0003400

**Published:** 2008-10-15

**Authors:** Sitao Wu, Yang Zhang

**Affiliations:** Center for Bioinformatics and Department of Molecular Bioscience, University of Kansas, Lawrence, Kansas, United States of America; University College London, United Kingdom

## Abstract

We developed a composite machine-learning based algorithm, called ANGLOR, to predict real-value protein backbone torsion angles from amino acid sequences. The input features of ANGLOR include sequence profiles, predicted secondary structure and solvent accessibility. In a large-scale benchmarking test, the mean absolute error (MAE) of the phi/psi prediction is 28°/46°, which is ∼10% lower than that generated by software in literature. The prediction is statistically different from a random predictor (or a purely secondary-structure-based predictor) with *p*-value <1.0×10^−300^ (or <1.0×10^−148^) by Wilcoxon signed rank test. For some residues (ILE, LEU, PRO and VAL) and especially the residues in helix and buried regions, the MAE of phi angles is much smaller (10–20°) than that in other environments. Thus, although the average accuracy of the ANGLOR prediction is still low, the portion of the accurately predicted dihedral angles may be useful in assisting protein fold recognition and *ab initio* 3D structure modeling.

## Introduction

There are three backbone dihedral torsion angles along with the protein peptide chains, which dictate the topology of protein 3D structures, i.e. ϕ (involving backbone atoms C-N-C_α_-C), ψ (N-C_α_-C-N), and ω (C_α_-C-N-C_α_). Because of the planarity of the partial-double peptide bond, the torsion angle ω is almost fixed at 180° with rare *cis* cases of 0° [1]. Therefore, if the values of the phi (ϕ) and psi (ψ) angles are known, the geometry of the global protein structures can be readily constructed with the standard bond length. The experimental procedure of the phi/psi angle determination is usually laborious and time-consuming. With the development of computing technology, the computer-based algorithms can accelerate the determination of backbone dihedral torsion angles. For example, SHIFTOR [2] and PRIDICTOR [3] developed at Wishart's lab can generate quickly high-resolution predictions of phi and psi values using the chemical shift data and the sequence information. In the field of structural bioinformatics, the torsion angle prediction data have found their usefulness in aiding secondary protein structure prediction [4], [5], sequence alignment [6], fold recognition [7], [8] and protein structure modeling [9], [10].

Encouraging progress has been made in purely sequence-based backbone torsion angle predictions, where investigators usually divide the backbone conformations into several discrete states based on the phi/psi values and then use various training algorithms to predict the states of variant phi/psi values [5], [7], [11]–[15]. The popular training techniques include neural networks (NN) [5], [14], support vector machines (SVM) [14], [15] and hidden Markov models (HMM) [7], [11]. Although these methods can achieve up to 80% prediction accuracy on the discrete states, they could not specify the real phi/psi values at each state, which renders the predictions less informative especially when the state division is rough. Wood and Hirst [4] first developed the DESCTRUCT algorithm which trains the sequence profile and the secondary structure information by neural networks to generate the continuous and real-value psi-angle predictions. The correlation coefficient between the predicted and experimental values is about 0.47. Later, Dor and Zhou [16] developed another neural network based program of SPINE which claimed a higher correlation coefficient of 0.62.

While both DESCTRUCT and SPINE trained their data on neural networks (NN), it is well-known that NN trains its parameters based on local optimization [17]. Compared with NN, SVM has the advantage of identifying the global optimum despite longer training time [18]. To further improve the phi/psi angle prediction accuracy, as well as to systematically examine the state-of-the-art of the dihedral angle predictions based on a large-scale protein set, we try to develop a new composite prediction tool using both NN and SVM techniques. Except for the sequence profiles obtained by PSI-BLAST [19], we found that the predicted secondary structure and solvent accessibility information can enhance the accuracy of the torsion angle predictions when used in a coherent training. The predictions are benchmarked on a large-scale set of non-redundant known proteins; these are also compared with the results of other algorithms in literature and the random angle predictions with the goal to systematically examine the strength and weakness of the algorithms at different environments.

## Methods

**Figure 1:**
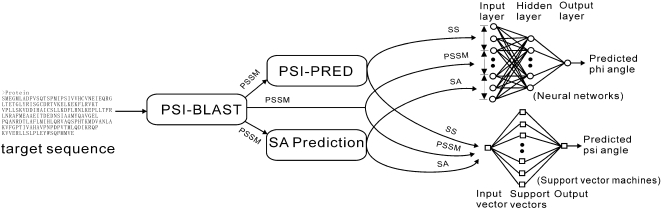
Flowchart of ANGLOR for the phi and psi angle predictions. Three sets of features, position-specific scoring matrix (PSSM), secondary structure (SS) and solvent accessibility (SA), are used as inputs of two machine-learning predictors (neural networks and support vector machines) for phi and psi separately.

The flowchart of ANGLOR is presented in Figure 1. For a given target sequence, ANGLOR first generates multiple sequence alignments by searching through a non-redundant sequence database. The sequence profile is then used to generate secondary structure and solvent accessibility predictions. Finally, all the features are fed into two machine learning tools (NN and SVM) with outputs being the predicted real-value phi and psi angles.

In the following, we introduce the prediction algorithms, training and testing data in details.

### Input features of training machines

For a given residue of the target protein, we extract three types of the sequence-based features: (1) position-specific scoring matrices (PSSM); (2) secondary structure (SS) predictions; (3) solvent accessibility (SA) predictions. The PSSM is generated by the PSI-BLAST search of the query against a non-redundant sequence database [19] with 20 log-odds scores taken at each position. The secondary structure (SS) is predicted by PSI-PRED [20], with the three states defined as alpha-helix, beta-strand, and coil. The solvent accessibility (SA) is predicted by the neural networks as well [21], [22], where a two-state feature is assigned to the residue *i* dependent on predicted SA values <25% (buried) or ≥25% (exposed).

The input features for residue *i* should include neighboring residues in a window around *i* since the phi and psi angles are strongly correlated with the structures of neighboring residues. We calculated the average prediction error of a simple SVM training (only with the PSSM feature) on 460 non-homologous validation proteins using different window sizes of 11, 13, …, 23. As a result, the window size of 21 is a suitable value with a low MAE value (a definition of MAE will be given below) and in the meantime with acceptable computer resource consumption.

To select an appropriate set of input training features, we tried different composition of predictors based on PSSM, PSSM+SA, PSSM+SS, and PSSM+SA+SS. We found that with the introduction of solvent accessibility (SA) into PSSM, the MAE value is decreased by 2% (or 5%) for phi (or psi) angles. With both SA and secondary structure (SS) added into PSSM, the MAE value is decreased by 6% (or 27%) for phi (or psi) angles. Therefore we select PSSM+SA+SS as our input feature set and the window size equal to 21 in our final training. The total number of the features in PSSM+SS+SA is 525 [ = 21*(20+3+2)] for the training of phi or psi angles.

### Training techniques: combination of NN and SVM

To find the most efficient training technique, we test both NN and SVM [23] as predictors for different angle predictions. For NN, we use the FANN software [24]. By trial and error, the best performance on the validation proteins is obtained by training with 50 hidden neurons in one hidden layer and 1000 epochs; the other parameters are used as given by default in FANN.

For SVM, we use the LIBSVM software [25] where the support vector regression is used instead of the support vector classification in comparison with other SVM tools. We obtain the least MAE on validation data by training with γ = 0.005 for radial basis kernel functions (data not shown); the other parameters are used by default in LIBSVM.

After the parameter optimization of each predictor, for phi angles on validation data, MAE by NN is 10% less than that by SVM. For psi angles, however, MAE by SVM is 10% less than that by NN. For the best performance, we will use FANN for the phi angle prediction and LIBSVM for the psi angle prediction. We also attempt to combine the consensus results of two predictors by voting; but it does not work as good as the best individual predictor in the phi/psi angle predictions (data not shown). We will discuss in more detail the difference of SVM and NN performance in the Result section.

### Training, validation and testing protein sets

For the training, validating and testing of the algorithms, we select 1,989 non-homologous proteins (<25% sequence identity) with size ranging from 50 to 865 from the PDB library through PDBSELECT (2006 March) [26], where the entries with broken chains or missing residues have been excluded. Among them, 500 (460/1,029) proteins are used as training (validation/testing) data. The total residues in the 500 (460/1,029) proteins are 72,918 (89,653/146,517). We use DSSP program [27] to extract the experimental values of the phi and psi angles. The phi/psi angles of the N- and C-terminal residues are neglected due to the incompleteness of four continuous backbone atoms. A list of the training, validation and testing proteins is available at our website http://zhang.bioinformatics.ku.edu/ANGLOR/benchmark.html.

### Evaluation criterion

Throughout the validation and testing of the algorithms, we assess the phi/psi angle predictions by the mean absolute error (MAE), which is defined as the average difference in degrees between the predicted (*P*) and the experimental values (*E*) of all residues, i.e. 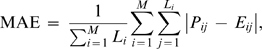
(1)where *M* is the number of proteins, *L_i_* is the total number of residues (excluding N- and C-terminals) in the protein *i*. Here, both *P* and *E* are in the range of [−180°, 180°]. A direct subtraction of the two values may result in an artificial MAE >180°. For example, when *P_ij_*  = −170° and *E_ij_* = 175°, the real prediction error should be 15° but the direct angle subtraction is 345°. To rule out the artificial effect, we make a transformation of the predicted angles before comparing them to *E* in Eq. 1, i.e.
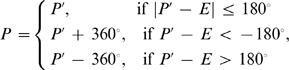
(2)where *P*′ is the original value of the predicted torsion angles.

Here, we do not use the Pearson correlation coefficient (CC) of *P* and *E* in our evaluation. Because of the angle transformation, the predicted phi/psi angles for some residues can go beyond the region [−180°, 180°]. Since the CC calculation is very sensitive to the outliers in the *P-E* plot, a small change of *P* in these residues may lead to drastic changes in the CC values. On the other hand, if we do not make the angle transformations, irregular correlation coefficient will be generated due to the artificial angle values near the border. These render CC a less robust quality assessment compared with MAE.

## Results

### Overall results

We calculate the average performance of the ANGLOR dihedral angle predictions for the 1,029 non-homologous testing proteins, which are also non-homologous to the training and validation proteins. The mean absolute errors, MAE, for all the 146,517 residues are 28.2° and 46.4° for phi and psi respectively.

**Figure 2:**
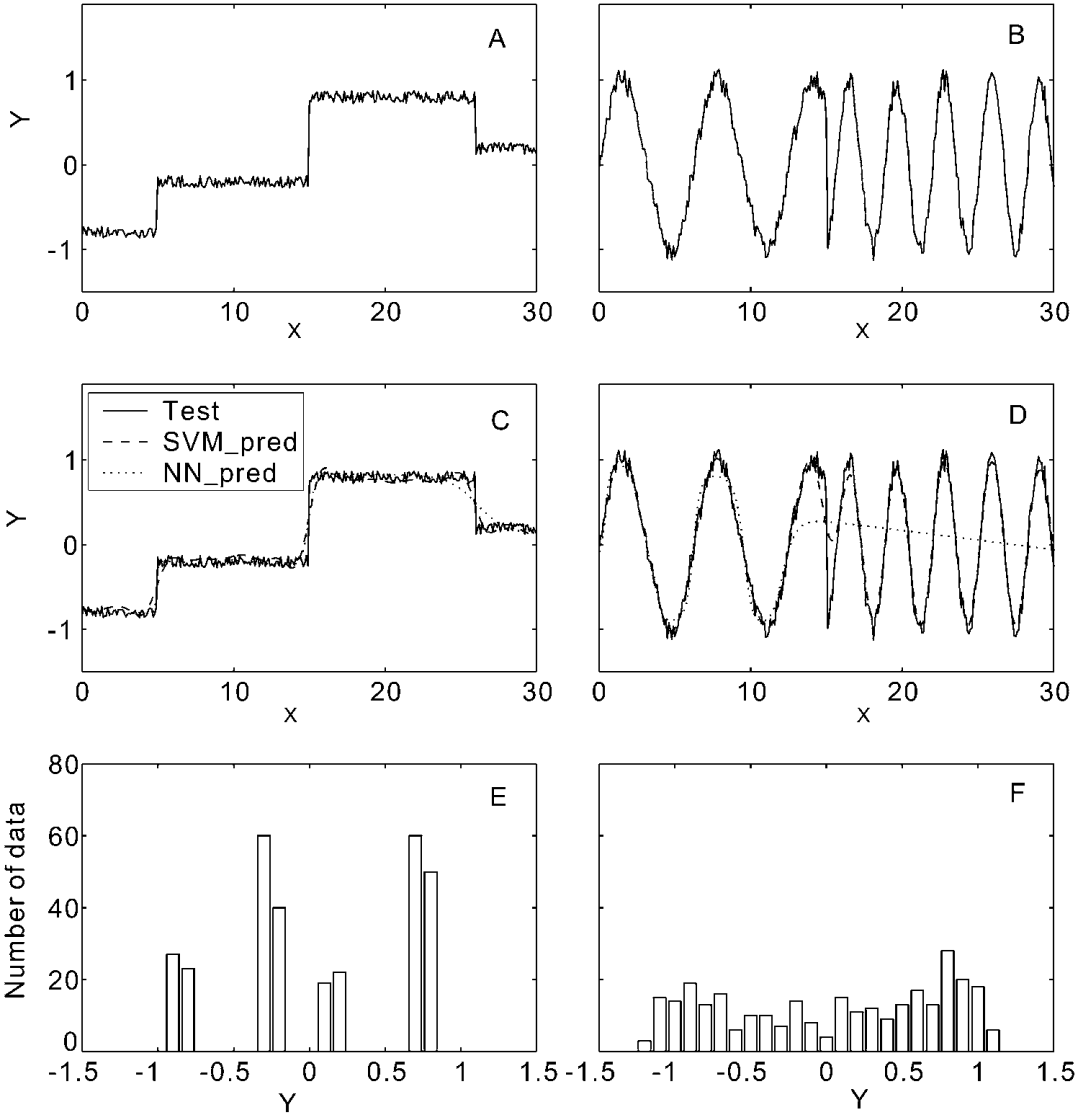
Two simplified models with the output *Y* generated from random processes for a given input *X* in [0, 30]. (A) Training data generated from the random fluctuations around four horizontal line segments; (B) training data generated from the random fluctuations around two sine waves of the frequency equal to 1/2π and 1/π respectively; (C) testing data (solid) and prediction results by two training predictors of SVM prediction (dashed) and NN prediction (dotted) for the model from A; (D) testing data (solid) and prediction results by two training predictors of SVM prediction (dashed) and NN prediction (dotted) for the model from B; (E) histogram of *Y* from A; (F) histogram of *Y* from B.

It is interesting to note that phi angle predictions are obviously more accurate than psi angle predictions, although the predictors have been trained based on the same set of proteins with the same set of features. To understand the mathematic reason behind the difference, we consider two simplified models as shown in Figures 2A and 2B. In Figure 2A, the values of *Y*-axis (output) are generated by random fluctuations around four constants in the specific regions of *X* (input), i.e.
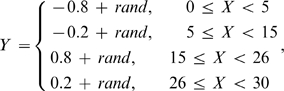
(3)where *rand* is a random number uniformly distributed in [−0.15, 0.15]. In Figure 2B, the *Y* values are generated with random fluctuations around two sine waves, i.e.
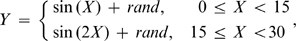
(4)where *rand* is the same as that in Eq. 3. The first function is obviously easier to predict by machine learning if the algorithm can find the ranges of four line segments, while in the second model the algorithm needs to recover two sine functions with different frequencies. Actually, when we use SVM (or NN) programs with the best tuned parameters to train these two models, the MAE of the *Y* prediction for the testing data is 0.069 (or 0.061) and 0.104 (or 0.369) for Models 1 and 2, respectively. The predicted *Y* values by the different techniques for the two models are presented in Figures 2C and 2D respectively. The best MAE (0.061) of Model 1 is 70% lower than that (0.104) of Model 2, which indicates Model 1 is indeed easier to predict.

For Model 1, the performance of NN is slightly better than that of SVM. It is because SVM tends to memorize all possible support vectors around the training curves which may be over-fitted for a simple function as Model 1. NN uses only 5 hidden neurons with less memorization and can achieve similar (or even better) performance for the simple patterns. For Model 2, a more complicated function, SVM memorizes all possible support vectors around the training curves in Figure 2B so that the prediction is close to testing data in all the range as shown in Figure 2D. However, NN uses unified weights for different input regions which is biased towards some specific input region, e.g. [0, 13] in this example (Figure 2D). The NN performance in the whole region is thus deteriorated for the more complicated curves. This difference may explain the reason for the performance variations of NN and SVM on phi and psi angles as seen in the training and testing data, because the psi angle distribution is more complicated.

To quantitatively assess the complexity of the models, we divide the outputs into *N* equally spaced bins and define the entropy of the models as
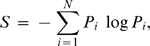
(5)where *p_i_* is the probability of *Y* in the *i*th bin. For the two models in Figure 2, the entropy of Figure 2A is 1.99, which is 46% lower than that of Figure 2B (2.91). The lower entry means that the model is less uncertain and therefore easier to learn.

A more intuitive way to view the uncertainty of the models is to plot the histogram of the outputs. If the output distributions are biased to some specific values as shown in Figure 2E, the entropy is lower and the model is thus easier to learn. On the other hand, if the output histogram tends to be uniformly distributed in a larger range as shown in Figure 2F, the entropy should be higher.

**Figure 3:**
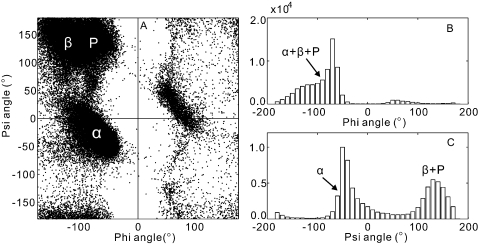
Ramachandran plot and histograms of phi and psi angles calculated from residues in 500 non-homologous training proteins. (A) Ramachandran plot; (B) histogram of phi angles; (C) histogram of psi angles. Alpha-helix, beta-strand and polyproline-II are represented by “α”, “β” and “P” respectively.

In Figure 3A, we present the Ramachandran plot which is collected from 500 training proteins, where the experimental phi values have only a single peak around −70° (corresponding to alpha-helix, beta-strand and polyproline-II in Figure 3B) while psi angles have two peaks around −50° (alpha-helix) and 130° (beta-strand and polyproline-II in Figure 3C) [28]. From a statistical perspective, the narrow single-peak distribution of phi angles and double peaks of psi angles in the Ramachandran plot result in the different degrees of uncertainty and therefore the different prediction accuracy for the phi and psi angles. Physically, the narrow distribution of the phi angle is due to the larger steric collision effect of the backbone oxygen atom when phi changes, compared to that of the hydrogen atom on N which corresponds to the psi angle change [1], [29]. More specifically, the entropy of the phi angles calculated from the 500 non-homologous proteins by using 36 bins in [−180° 180°] is 2.67, which is 13% less than that of psi angles (3.03).

As a comparison, we also calculate the prediction accuracy for psi angles by SPINE (which provides only psi angle predictions), based on the same set of testing proteins. We note that the testing proteins are not necessary to be non-homologous to the SPINE training proteins because the list of training proteins is not available to us. We are unable to show the data from DESCTRUCT here because we do not have the software or find its online server. Overall, ANGLOR has a clearly higher accuracy (with MAE = 46.4°) than SPINE (with MAE = 50.9°). The better performance of ANGLOR may be due to the optimized combination of both NN and SVM training techniques and the more training features (PSSM+SA+SS). The selection of the angle range of training is also different in our algorithm (while SPINE [16] uses a nonlinear angle-transformation in their training which we found harmful to the accuracy in our case).

**Figure 4:**
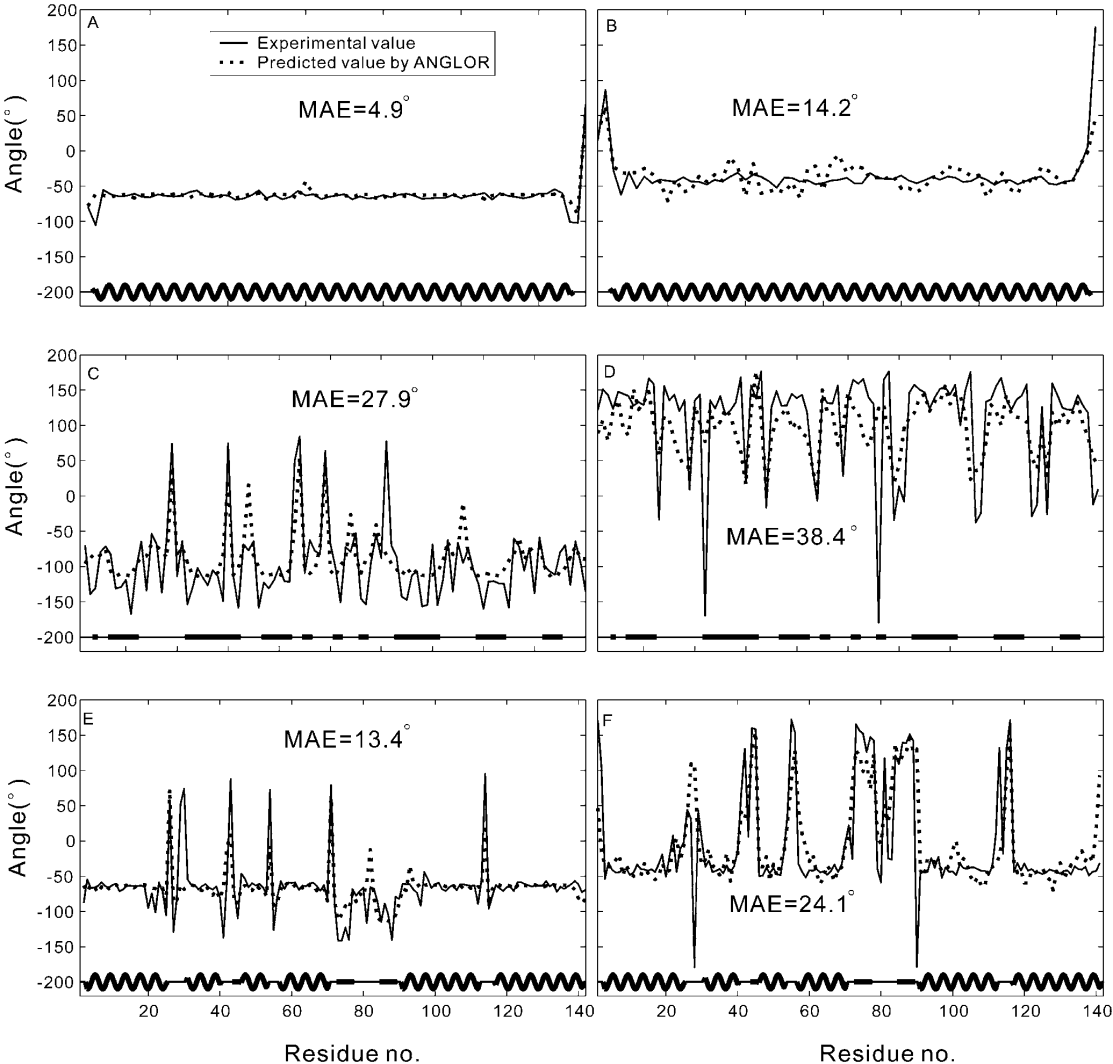
The comparison of predicted (dotted lines) and experimental values (solid lines) of phi and psi angles for three typical alpha-, beta-, and alpha/beta-proteins. Secondary structures of the proteins are signified at the lower part of each box, with coil, beta-strand, and alpha-helix residues represented by thin lines, thick lines, and thick curves, respectively. (A) phi angle for 1n7sD; (B) psi angle for 1n7sD; (C) phi angle for 1k5nB; (D) psi angle for 1k5nB; (E) phi angle for 1lj9B; (F) psi angle for 1lj9B.

In Figure 4, we show illustrative examples of the ANGLOR prediction on phi and psi angles from three typical alpha-, beta-, and alpha/beta-proteins. The first protein is from Chain D of the truncated neuronal snare complex protein (PDB ID: 1n7s), which has 66 amino acids and includes one long alpha helix (Figures 4A and 4B). The predicted phi angles are close to experimental value with a MAE = 4.7° for the alpha residues and a MAE = 7.6° for the coil ones. In the second example, the target, from Chain B of human leukocyte antigen (PDB ID: 1k5n), has 100 residues and contains ten beta strands. Compared to the alpha proteins, the prediction accuracy is lower for both phi (MAE = 24.1° for beta residues and MAE = 31.7° for coil residues) and psi (MAE = 26.8° for beta residues and MAE = 49.7° for coil residues) angles. But the ANGLOR predictions still follow well the experimental curves (Figures 4C and 4D). In the third example, we show the alpha/beta-protein from Chain B of the transcriptional regulator protein (PDB ID:1lj9) which has 142 residues with six alpha helices and three beta strands. The overall prediction accuracy is between the alpha and beta proteins, i.e. for phi (psi) angles, MAE = 6.3° (15.1°) for alpha residues, MAE = 22.8° (25.0°) for beta residues, and MAE = 36.0° (57.8°) for coil residues.

### Residues in different environments

In Table 1 (Rows 4–6), we present the ANGLOR prediction data for the residues from different secondary structures. In general, it is known that the local geometry of coils has much higher diversities than that of the regular secondary structures. Accordingly, the MAE value of the phi/psi angles in our predictions is much smaller in regular secondary structures than that in coil regions. Moreover, the MAE value of the alpha-helix residues is smaller than that of the strands, which is also closely related with the complexities of the angle distributions. Quantitatively, the alpha-helix residues have the lowest angle entropy for phi (1.60) and psi angles (1.74) while the coil residues have the largest angle entropy (2.92 and 3.28) (see Columns 5 and 8 in Table 1).

**Table 1:** Comparison of ANGLOR with random predictor in different environments.

		Phi angle			Psi angle		
		MAE_ANGLOR_ 4	MAE_random_	Entropy	MAE_ANGLOR_	MAE_random_	Entropy
All		28.2°±0.1°	49.3°±0.1°	2.67	46.4°±0.1°	88.8°±0.2°	3.03
SS^1^	Helix	11.0°±0.1°	39.0°±0.2°	1.60	28.2°±0.1°	85.1°±0.3°	1.74
	Strand	27.9°±0.1°	50.0°±0.2°	2.46	39.9°±0.2°	93.4°±0.4°	2.35
	Coil	41.8°±0.2°	57.1°±0.2°	2.92	63.9°±0.2°	89.4°±0.2°	3.28
SA^2^	Buried	24.1°±0.1°	46.8°±0.2°	2.59	41.5°±0.1 °	89.7°±0.3°	2.90
	Exposed	31.2°±0.1°	51.2°±0.2°	2.71	49.9°±0.2°	88.2°±0.2°	3.10
AA^3^	ALA	22.5°±0.3°	45.0°±0.4°	2.36	42.7°±0.4°	88.3°±0.6°	2.76
	ARG	25.0°±0.4°	46.1°±0.5°	2.53	44.1°±0.5°	88.5°±0.8°	2.91
	ASN	37.6°±0.5°	54.9°±0.6°	2.77	45.9°±0.5°	87.3°±0.7°	3.24
	ASP	30.8°±0.4°	48.3°±0.5°	2.61	48.9°±0.5°	87.6°±0.7°	3.16
	CYS	27.7°±0.6°	47.4°±0.8°	2.67	48.7°±0.8°	89.7°±1.2°	3.05
	GLN	25.1°±0.4°	46.4°±0.6°	2.49	43.0°±0.5°	88.0°±0.9°	2.85
	GLU	23.3°±0.3°	44.9°±0.4°	2.44	43.1°±0.4°	87.8°±0.7°	2.83
	GLY	75.1°±0.5°	94.1°±0.6°	3.17	66.9°±0.5°	89.0°±0.5°	3.34
	HIS	31.8°±0.6°	49.6°±0.7°	2.68	48.2°±0.7°	88.6°±1.1°	3.10
	ILE	18.1°±0.2°	43.0°±0.4°	2.39	35.3°±0.4°	89.4°±0.7°	2.67
	LEU	18.3°±0.2°	42.2°±0.4°	2.38	38.1°±0.3°	88.4°±0.6°	2.79
	LYS	25.6°±0.3°	46.6°±0.4°	2.58	45.6°±0.4°	88.2°±0.7°	2.95
	MET	22.4°±0.5°	45.2°±0.8°	2.48	40.9°±0.7°	88.7°±1.2°	2.87
	PHE	24.4°±0.3°	46.7°±0.5°	2.57	40.8°±0.5°	89.6°±0.9°	2.94
	PRO	15.2°±0.2°	38.2°±0.5°	1.61	61.3°±0.5°	90.3°±0.8°	2.91
	SER	32.3°±0.4°	49.2°±0.4°	2.67	55.4°±0.4°	89.5°±0.7°	3.04
	THR	26.0°±0.3°	45.8°±0.4°	2.57	51.1°±0.5°	90.0°±0.7°	2.99
	TRP	23.1°±0.5°	45.1°±0.9°	2.56	43.5°±0.8°	89.5°±1.4°	2.88
	TYR	25.3°±0.4°	47.2°±0.6°	2.56	42.3°±0.6°	89.7°±0.9°	2.94
	VAL	20.1°±0.2°	44.5°±0.4°	2.46	37.6°±0.4°	90.0°±0.7°	2.70

1 SS: residues in different secondary structure

2 SA: residues of different solvent accessibility

3 AA: 20 amino acid types

4 MAE±standard error of mean

In Rows 7 and 8, we divide the predictions into buried and exposed categories, where the buried residues are defined as those with a relative solvent accessible area <25% and other are exposed residues (a definition with other SA cutoffs is possible but will result in similar results). The buried residues have a MAE (for phi/psi angles) which is 29%/20% lower than the exposed residues in the ANGLOR prediction. This is due to the fact that the angle entropy of the buried residues is lower than that of the exposed ones, demonstrating the higher regularities of the protein fragments in core regions.

Due to the various steric collisions between the side-chain and the main-chain [1], it is anticipated that different amino acids have different entropies and thus different degrees of difficulties for the torsion angle predictions. In the lower part of Table 1, we examine the ANGLOR performance for each of 20 amino acids. Not surprisingly, Glycine has the largest prediction error (75°/67° for phi/psi angles), which is mainly due to the fact that Glycine has no side-chain atom except for a proton. It has therefore the least steric restriction to the backbone dihedral angle motions. Accordingly, the angle entropy of Glycine is the highest among all the 20 amino acids (Table 1).

Proline has the least MAE (∼15°) for phi angles but has an unusually large MAE (∼61°) for psi angle prediction. This is because of its special side-chain structure which has the delta-carbon atom attached to the backbone nitrogen and significantly restricts the backbone rotation at the phi direction. But the leaning of side-chains toward the nitrogen has almost no steric restriction to the C-O backbone atoms. These result in a significant difference in the torsion angle entropy for Proline between phi (1.61) and psi (2.91).

### Comparison to naïve predictors

Although the above comparisons show some degree of advantage of ANGLOR over other algorithms in literature, a comparison of the ANGLOR prediction to simple and naïve predictors should help to quantitatively justify the necessity of the training efforts.

We first compare ANGOR with a naïve random predictor. A simple method to generate the random prediction is to take phi/psi angles randomly from an evenly distributed pool in [−180°, 180°], which will have an average MAE = 90°. In an alternative way, we randomly take the phi/psi angles from an amino-acid-specific pool that is collected from 500 training PDB proteins. Since the pool has the information of angle distribution of real PDB structures, the second method should generate more accurate angle predictions than the first method. In the following, we will compare ANGLOR with the second (more challenging) data set. To have a stable distribution, the random process is repeated by 10,000 times for each target residue.

For the 1,029 testing proteins, the performance (MAE) of all residues in specific local environments by the random predictor is listed in Columns 4 and 7 of Table 1. Overall, the ANGLOR prediction is better than the random prediction with MAE reduced by 21.1° for phi and by 42.4° for psi. For the residues in different secondary structures, the improvement for alpha-helix residues is the largest (by 28.0° for phi and by 56.9° for psi) despite of the fact that the random prediction for alpha-helix residues in our sample is the lowest. This indicates that the machine-learning techniques work the best for those residues which have the best regularities. If we look at the specific amino acids, the MAE of the ANGLOR prediction is significantly smaller than that of the random predictor for all amino acid types with *p*-values <1.0×10^−73^ by Wilcoxon signed rank test. The overall *p*-value when counting all amino acids is close to zero (<1.0×10^−300^).

Second, we compare ANGOR with a more challenging predictor based only on secondary structure predictions. For this purpose, we first calculate the average torsion angles in three secondary structures (α helices, β strands and coils) by DSSP program [27] on the 500 training proteins with solved 3D structures, i.e. phi(psi) = −64.7°(−37.6°) for helices, −111.0°(122.2°) for strands, and −67.3°(55.0°) for coils. Then for test data, we predict the secondary structure status of each residue by PSI-PRED [20] with the phi/psi angles given by the mean values calculated from the statistics of the PDB structures. Using this simple predicator, the overall accuracies for the 1029 testing proteins are MAE = 30.4° for phi angles and MAE = 49.6° for psi angles, which are (not surprisingly) much higher than the random predictions. But ANGLOR predictions are still 2.2° more accurate in phi angle and 3.2° more accurate in psi angle than the naïve secondary- structure-based predictor. This difference corresponds to a *p*-value <1.0×10^−148^ by Wilcoxon signed rank test. The increase in accuracy shows the adding to a purely secondary-structure-based predictor by the combinatory training of SS, PSSM and the solvent accessibility information.

## Discussion

We developed a composite machine-learning algorithm of ANGLOR for *ab initio* prediction of real-value backbone torsion angles (phi and psi), which has been tested on the large-scale non-homologous protein set. One of the main purposes of this work is to examine systematically the state-of-the-art of the machine-learning based dihedral angle predictions and estimate the potential usefulness in 3D structure predictions. The executable ANGLOR program and the on-line server are freely available for academic users at http://zhang.bioinformatics.ku.edu/ANGLOR.

Technically, we found that the current phi/psi prediction can be further improved by the combination of different training methods and a more comprehensive selection of input features. By using SVM for psi angles and NN for phi angles which use features including sequence profiles, predicted secondary structures and solvent accessibilities, the mean absolute error (MAE) of the ANGLOR psi angle predictions is >10% smaller than that of the available software in literature. As a confirmation of the necessity of the training, the MAE of ANGLOR is statistically smaller than those of a purely secondary-structure based predictor and a random predictor with a *p*-value <1.0×10^−148^ and <1.0×10^−300^ by Wilcoxon signed rank test, respectively.

The accuracy of the machine-learning based torsion angle predictions is closely related with the diversities of the angle distributions in real structures. In general, the psi angles are more divergently distributed than phi and therefore the phi prediction is more accurate than that of psi. Similarly, ANGLOR generates better predictions for the residues in helices/strands than those in coils, and for buried residues than exposed residues. By the analysis of two simplified models, it is shown that the entropy can be used to quantitatively define the angle distribution diversities, which is closely correlated with the machine-learning performance.

Because of the various steric collision effects of side-chain with backbone atoms, different amino acids have different degrees of freedoms in the backbone torsion angles. This results in much lower prediction accuracy of ANGLOR for some flexible amino acids than others. For example, both MAE for phi/psi of Glysine and for psi of Proline are >60°. One way of improving ANGLOR in future is to develop additional predictors specifically trained for the phi and psi angles of Glysine, as well as split Proline into specific (trans-, cis-, down- and up-) conformations. On the contrary, some other residues (ILE, LEU, PRO, VAL) have much higher accuracy than the average.

Overall, the accuracy of phi and psi angle predictions by ANGLOR (with a MAE of 20–45°) is still too low to reconstruct a meaningful 3D model directly from the predictions. Nevertheless, it may be possible to exploit the predictions as loose restraints to guide the fold-recognition and *ab initio* simulation procedures. We have recently combined the phi and psi angle predictions into a profile-profile alignment algorithm [30], where the input features for the angle prediction are similar as ANGLOR but both phi and psi predictions were trained by SVM [8]. It was found that the average TM-score [31] of the first identified templates can be increased by 2.5% with the introduction of the torsion angle restraints, where the difference between ANGLOR-predicted angles and the experimental angles in templates was added to the profile-profile alignment scores. If coupled with additional features of solvent accessibility and hydrophobic scoring matrix, the TM-score improvement can be increased up to 5% [8].

We are also working on incorporating the ANGLOR prediction into the I-TASSER simulation [22], [32] for *ab initio* protein structure modeling, where the dihedral angles are used as restraints to guide the local backbone movements. Although the average MAE of the phi and psi angles is big, the phi angle predictions from some specific residues (e.g. ILE, LEU, PRO, VAL) and in some specific environments (e.g. helix regions), which have smaller MAE, should be chosen. The work is still in progress when this paper is prepared.
